# Visceral Adipose Tissue Alters Podometrics and Renal Compensation After Uninephrectomy

**DOI:** 10.1016/j.ekir.2025.103739

**Published:** 2025-12-23

**Authors:** Christopher Paschen, Maximilian C. Koeller, Juliane Hennenberg, Johannes Kläger, Maja Nackenhorst, Andre Oszwald, Michael Kammer, Nicolas Kozakowski, Dietmar Tamandl, Heinz Regele, Rainer Oberbauer

**Affiliations:** 1 Division of Nephrology and Dialysis, Department of Medicine III, Medical University of Vienna, Vienna, Austria; 2 Department of Pathology, Medical University of Vienna, Vienna, Austria; 3 Department of Biomedical Imaging and Image-guided Therapy, Medical University of Vienna, Vienna, Austria; 4 Center for Medical Data Science, Institute of Clinical Biometrics, Medical University of Vienna, Vienna, Austria

**Keywords:** deep learning, kidney morphometry, nephrectomy, podometrics, visceral fat

## Abstract

**Introduction:**

Obesity is an established risk factor for chronic kidney disease (CKD). However, excess visceral adipose tissue (VAT) termed visceral obesity (VO) can occur in individuals with normal body mass index (BMI) or overweight. VO is associated with impaired kidney function but its effect on kidney morphology remains unclear. This study aimed to examine the association of VO with glomerular ultrastructure, podocyte morphometry (podometrics), and the kidneys’ ability for compensation after uninephrectomy in normal BMI and overweight individuals.

**Methods:**

VAT was retrospectively quantified in computed tomography (CT) of 52 patients (BMI < 30 kg/m^2^) who underwent nephrectomy for nonmetastatic renal tumor without previous chemotherapy or immunotherapy. VO was defined as VAT area ≥ 100 cm^2^. Histological sections from nontumorous kidney regions were examined using deep learning–supported glomerular morphometry and podometrics (podocyte count, density, and nuclear volume). Renal compensation in the first year after nephrectomy (change in estimated glomerular filtration rate [ΔeGFR]) was assessed using linear regression.

**Results:**

Of the 52 subjects with normal BMI or overweight, 35 were diagnosed with VO and exhibited a larger glomerular volume (2.6 ± 0.7 vs. 2.0 ± 0.5 ×10^6^ μm^3^; *P* = 0.004), lower podocyte density (194 ± 50 vs. 243 ± 59 per 10^6^ μm^3^; *P* = 0.003), and podocyte nuclear hypertrophy (226 ± 27 vs. 195 ± 22 μm^3^; *P* < 0.001). VO was associated with impaired eGFR compensation after uninephrectomy (ΔeGFR: −24 ± 15 vs. −12 ± 12 ml/min per 1.73 m^2^, *P* = 0.03). Structural changes, including glomerular enlargement (*P* = 0.005), podocyte density (*P* = 0.01), and nuclear hypertrophy (*P* = 0.003), were significantly associated with reduced ΔeGFR.

**Conclusion:**

VO was associated with glomerular and podocyte changes, and impaired kidney function compensation after nephrectomy in normal BMI and overweight individuals. These data suggest that VAT quantification could guide individual decision making in subjects planned for nephrectomy.

## Graphical abstract

**Figure:**
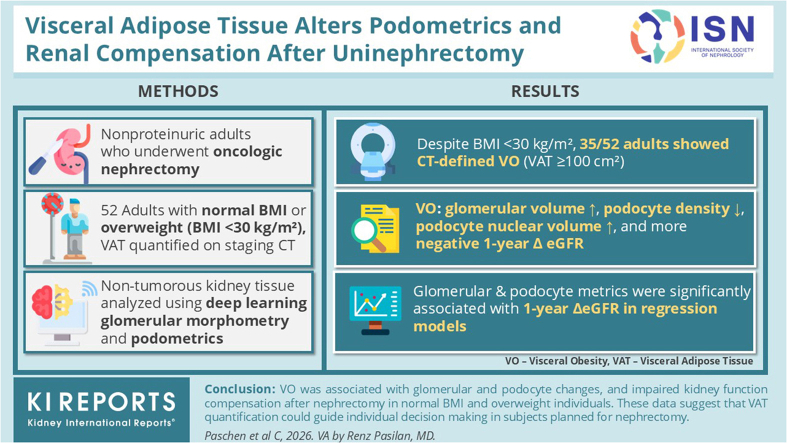

Obesity is an increasing public health concern and a risk factor for CKD.^1^^,^^2^ Structural changes associated with excess body weight include enlarged glomerular volume, podocyte loss, glomerulosclerosis, and tubular alterations.^2^, ^3^, ^4^ Especially, podocyte depletion can lead to further glomerulosclerosis, nephron loss, and is associated with progressive CKD in obesity-related glomerulopathy.^4^

Although BMI is an easily accessible measure for risk stratification, its explained variability for eGFR loss is low and furthermore, it does not provide an estimate on body fat distribution.^2^^,^^5^^,^^6^ VAT accumulates in the abdominal cavity and provides additional information about metabolic risk across BMI-defined weight categories.^5^^,^^7^^,^^8^ The excess of VAT is referred to as VO. VO is a BMI-independent risk factor for both the onset of new kidney disease and the progression of CKD, even in individuals with normal weight.^7^, ^8^, ^9^, ^10^

To our best knowledge, the previous literature on VAT-related histopathological findings in nonobese persons is scarce and reports increased percentage of glomerular sclerosis, interstitial fibrosis, and tubular atrophy in indication biopsies of individuals with prevalent CKD.^11^ Previous studies have primarily examined the relationship between BMI and glomerular alterations.^3^^,^^12^ In addition, research on the effect of excess body weight on podocyte morphometry (podometrics) has mostly been limited to patients with histologically proven obesity-related glomerulopathy.^3^^,^^4^ Further investigations into podocyte structure have examined individuals with elevated BMI; however, these studies primarily focused on diabetes, hypertension, or progressive CKD.^13^, ^14^, ^15^, ^16^, ^17^ Although an impact of VO on glomerular structure was suggested, the effect of VO on podometrics in normal BMI and overweight persons, who were not recruited for kidney disease, remains unknown.^11^

The aim of this study was to examine normal BMI and overweight individuals for the prevalence of VO and to investigate the association of VO with changes in glomerular and podocyte structure. A secondary goal was to test whether morphometric parameters are associated with impaired kidney function compensation among subjects undergoing nephrectomy for a renal tumor.

## Methods

### Population

**Figure 1:**
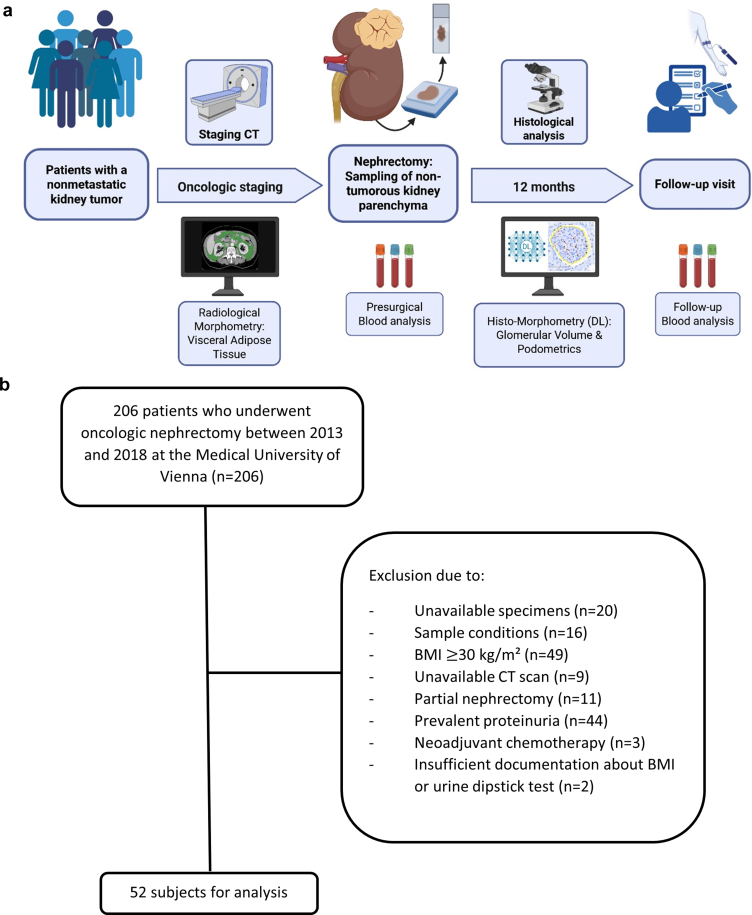
Flow chart of the study procedures is presented in Panel A. The cohort consists of normal BMI and overweight patients with a nonmetastatic kidney tumor who underwent tumor nephrectomy. Presurgical routine foresees blood and urine sampling as well as computed tomography (CT) for oncologic staging. CT images were morphometrically examined for visceral adipose tissue. The nontumorous kidney parenchyma of the nephrectomy specimens was first examined for histopathological scoring and subsequently for the deep-learning-based morphometry of glomeruli and podocytes. A follow-up visit including a blood sampling was performed after 12 months. Panel B presents the study flow chart detailing patient eligibility and sample size. Panel A was created in BioRender. Paschen, C. (2025) https://BioRender.com/7sy2hok. BMI, body mass index.

We enrolled nonproteinuric subjects who underwent oncologic nephrectomy between 2013 and 2018 at the Medical University of Vienna. Subjects with a presurgical BMI < 30 kg/m^2^ were included in the study. Clinical documentation had to include presurgical CT for oncologic staging. Further, patients with prevalent proteinuria, or previous neoadjuvant chemotherapy were not included in this study. Absent proteinuria was confirmed through negative urinary dipstick analysis. Sex was defined as biological sex (male or female) as recorded in the medical record. Arterial hypertension was assessed through clinical documentation or an established blood pressure–lowering therapy. The study procedures and the patient flow chart are displayed in Figure 1. The completed STROBE checklist is provided in Supplementary Table S1. The institutional review board of the Medical University of Vienna approved this study (EK: 2107/2021). The study was conducted in accordance with the Declaration of Helsinki.

### VAT, Waist Circumference, and Kidney Volumetry

**Figure 2:**
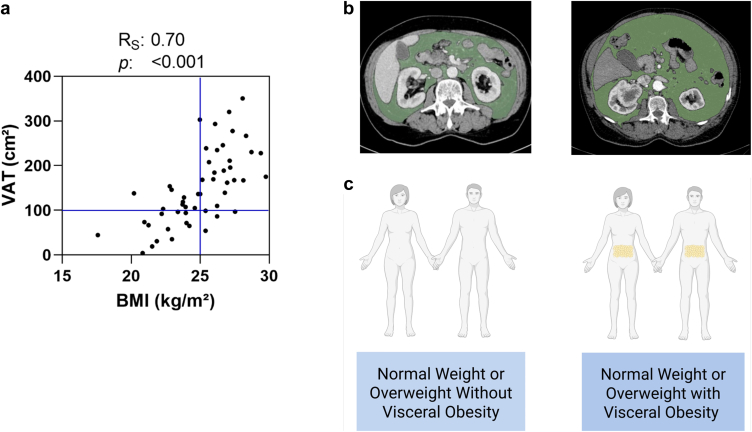
Panel A displays a scatter plot illustrating the distribution of body mass index (BMI) and visceral adipose tissue (VAT). The vertical line marks the BMI threshold for normal weight and overweight (25 kg/m^2^), while the horizontal line represents the threshold for visceral obesity (VO – defined as VAT area ≥ 100 cm^2^). VAT measurements on contrast-enhanced computed tomography scans are shown in Panel B. The analysis was performed on an axial section at the third lumbar spine level. VAT was identified within a density range of −150 to −50 Hounsfield units and manually corrected. Panel B depicts an individual without VO (VAT < 100 cm^2^) on the left, whereas an individual with VO (VAT ≥ 100 cm^2^) is shown on the right. Panel C presents visualization of individuals without and with VO. Panel C was created in BioRender. Paschen, C. (2025) https://BioRender.com/qgluxfx.

The venous phase of contrast-enhanced staging CT scans was examined using Horos, a free, open-source software for medical image viewing and analysis (version 3.3.6). VAT area was determined on the level of the third lumbar spine (Figure 2), which correlates with the total volume of the VAT compartment.^18^ The sections were selected through visibility of both transverse processes of L3.^19^^,^^20^ The “grow region” function was employed to annotate regions for VAT (Hounsfield units: −150 to −50) within the abdominal cavity.^19^ Resulting annotations were manually refined. In the next step, the “closing” function (2 pixels) was applied. The final annotations have been reviewed and approved by an experienced radiologist. VO was defined as ≥ 100 cm^2^ VAT area in concordance with previous reports on VO.^7^^,^^21^^,^^22^

Waist circumference was estimated at the L3 level by outlining the truncal cross-section with a polygon. Volumes of the contralateral, nontumorous kidney were determined using manual delineation of the renal contours on consecutive axial CT sections. Total kidney volume was computed by summing all cross-sectional areas and multiplying the result by the corresponding section thickness.

### Sample Acquisition and Staining

Histological specimens were acquired from tumor-free regions of the kidney. The nonaffected kidney parenchyma was examined as part of the clinical routine. In the histopathological routine workup, semiquantitative scoring was performed for mesangial expansion, intimal fibrosis, interstitial fibrosis, tubular atrophy, and arteriolar hyalinosis.^16^^,^^23^ Each lesion was classified as absent, mild (°I), moderate (°II), or severe (°III).^16^^,^^23^

**Figure 3:**
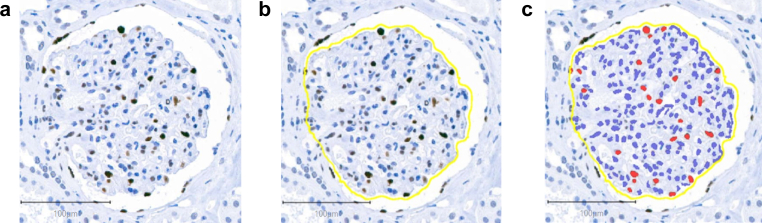
Panel A presents a photomicrograph of glomeruli from a nephrectomy specimen, immunostained with a nuclear Wilms tumor 1 (WT1) antibody. Panel B displays a segmented glomerular profile, which was processed using a deep learning–based segmentation algorithm, whereas Panel C shows the detected podocyte nuclei, whose diameters were extracted for podometrics.

For this study, formalin-fixed paraffin-embedded tissue blocks were dearchived and fresh 2 μm sections were cut for further processing. Sections were deparaffinized in xylene substitute (Neo-Clear, Sigma-Aldrich), rehydrated, and subjected to antigen retrieval in citrate buffer (pH: 6.0) by autoclaving for 20 minutes. Endogenous peroxidase activity was blocked with 3% hydrogen peroxide. Immunohistochemical staining of podocyte nuclei (Figure 3) was conducted using a nuclear Wilms tumor 1 protein antibody (abcam 89901, dilution 1:500, overnight at 4 °C). Detection was performed using a polymer-based secondary antibody system (Epredia Lab Vision UltraVision LP Detection System), followed by DAB chromogenic development for 120 seconds. Slides were counterstained with Mayer’s hemalum, dehydrated, and permanently mounted with Eukitt (O. Kindler GmbH). Slides were digitized with a 3D Histech Pannoramic 250 Slidescanner.

### Morphometrical Glomerulus Analysis

Nonsclerotic glomeruli were identified on a single histological section through a deep learning–based segmentation algorithm (global accuracy: 99.5%).^16^ QuPath (v0.3.2) and Microscopy Image Browser (v2.83) were used for glomerular tuft segmentation, excluding Bowman’s capsule and parietal epithelial cells.^24^, ^25^, ^26^ Predicted annotations were manually refined in QuPath.^16^ Glomerular volume was estimated using the method of Weibel and Gomez (β: 1.38; size distribution coefficient: 1.01)^12^^,^^27^ as follows:

$$Glomerular\;volume={Mean\;glomerular\;profile\;area}^{1.5}\times \frac{1.38}{1.01}$$

Positive cell detection was employed on segmented glomerular tufts to identify Wilms tumor 1–stained podocyte nuclei (Figure 3).^25^ Podocyte density was determined using the stereological single section method.^28^ The apparent nuclear caliper diameter, *d* was estimated from the polygons of Wilms tumor 1 positive nuclei.^16^^,^^28^ The true section thickness (T) was measured on a Zeiss Axio Imager 2 microscope using 100× oil immersion microscopy objective by recording the Z-axis distance between the uppermost and lowermost in-focus planes of the tissue.^16^ Measurements were performed in 3 randomly selected regions on each specimen (cohort mean true section thickness: 4.54 ± 0.23 μm), and the mean value of T for each individual was used to calculate podocyte density. Assuming a spherical shape of podocyte nuclei (shape coefficient: k = 0.72), apparent *d* was corrected with the section thickness to the true nuclear caliper diameter *D* with the following quadratic formula^28^:

$$D=\frac{d-T+\sqrt{{\left(d-T\right)}^{2}+4kdT}}{2k}$$

Podocyte density estimation necessitates a correction factor (CF) for the detected podocyte nuclei on a histological section^28^ as follows:

$$CF={\left(\frac{D}{T}+1\right)}^{-1}$$

Podocyte density was calculated using the equation of the single section method^28^ as follows:

$$Podocyte\;Density=\frac{Detected\;podocyte\;nuclei\times CF}{T\times Total\;tuft\;area\;per\;slide}$$

Multiplying podocyte density and glomerular volume estimated podocyte number per glomerular tuft. Podocyte nuclear volume was calculated stereologically^16^^,^^29^ as follows:

$$Podocyte\;nucleus\;volume=\frac{4\pi }{3}\times {\left(\frac{2}{\pi }\times d\right)}^{3}$$

Total podocyte nuclear volume per glomerulus was assessed by multiplying podocyte number by nuclear volume.^16^

### Statistical Analysis

Values are displayed as means and SD for normally distributed variables, and as medians and interquartile ranges for nonsymmetrically distributed variables. The distribution of each variable was assessed graphically (exemplarily displayed in Supplementary Figure S1 for morphometric parameters of the entire cohort).

Intergroup differences of continuous variables between individuals with and without VO were examined using *t* test. Before group comparisons, homogeneity of variances was assessed using Levene’s test, and in case of a significant result a *t* test for unequal variances was used. An analysis of covariance with BMI and age as covariables was performed as sensitivity analysis for intergroup comparisons. This was followed by propensity score matching (1:1 nearest neighbor) based on BMI, with BMI differences between groups compared using an independent samples *t* test. A further nonparametric sensitivity analysis was performed using Wilcoxon rank sum test. Ordinally scaled histopathological scores were compared using Wilcoxon rank sum test. Nominal data were compared using the χ^2^-test. Renal compensation was measured as ΔeGFR (difference from the prenephrectomy baseline to the 1-year follow-up) and relative ΔeGFR (ratio of ΔeGFR and prenephrectomy baseline eGFR). If follow-up data were not available, subjects were not included in the ΔeGFR analysis (2 subjects died shortly after surgery, 5 subjects were lost to follow-up after 1 month). Multiple linear regression analysis was used to assess the association between structural parameters and ΔeGFR, using VAT, and BMI as adjustment variables in model 1; VAT, prevalent hypertension, diabetes, age, and sex in model 2; and height-adjusted nonaffected kidney volume and baseline eGFR in model 3; and histopathological scores in model 4. All variables were standardized to mean 0 and unit variance for regression analysis. Residual diagnostics for regression (residuals vs. fitted values, normality of residuals) were assessed graphically. A 2-sided *P*-value < 0.05 was regarded as statistically significant; no correction for multiple testing was applied. Analyses were performed using SPSS (version 21; IBM Corp., Armonk, NY, USA) and GraphPad Prism (version 8; GraphPad Software, La Jolla, CA, USA).

## Results

### Patient Characteristics

The analysis comprised 52 nonproteinuric normal BMI and overweight subjects who underwent tumor nephrectomy and had an available presurgical staging CT scan (Figure 1). Demographic characteristics are presented in Table 1. The average age was 66 ± 13 years. All individuals tested negative for proteinuria on urinary dipstick. Mean BMI was 25 ± 3 kg/m^2^. The median VAT was 137 cm^2^ (interquartile range: 93–205 cm^2^). The criteria of VO were fulfilled in 35 individuals (67%), including 46% of individuals with normal weight and 86% of those with overweight (Figure 2). Most individuals with VO were men (77%), whereas most non-VO subjects were women (88%; *P* < 0.001). The prevalence of hypertension was higher in individuals with VO than in those without VO (74% vs. 47%; *P* = 0.053). Ten participants in the VO group had diabetes, whereas none of the participants in the non-VO group were affected (29% vs. 0%; *P* = 0.01).Total cholesterol and low-density lipoprotein levels did not differ between individuals with and without VO, respectively (total cholesterol: 185 ± 50 vs. 199 ± 38 mg/dl, *P* = 0.33; low-density lipoprotein: 109 ± 44 vs. 117 ± 34 mg/dl, *P* = 0.54), whereas VO was associated with lower high-density lipoprotein (50 ± 14 vs. 64 ± 21 mg/dl, *P* = 0.01). Individuals with VO exhibited a trend to higher triglyceride levels (129 ± 51 vs. 102 ± 50 mg/dl, *P* = 0.09).

**Table 1:** Baseline demographics

Characteristics	Non-VO (*n* = 17)	VO (*n* = 35)	*P*
Sex (female) – *n* (%)	15 (88%)	8 (23%)	< 0.001^a^
Age – yrs	60 (± 14)	69 (± 13)	0.03^a^
Arterial hypertension – *n* (%)	8 (47%)	26 (74%)	0.053
Diabetes mellitus – *n* (%)	0 (0%)	10 (29%)	0.01^a^
Weight – kg	63 (± 9)	77 (± 9)	< 0.001^a^
Height – m	1.65 (± 0.08)	1.72 (± 0.08)	0.01^a^
BSA – m^2^	1.7 (± 0.1)	1.9 (± 0.1)	< 0.001^a^
BMI – kg/m^2^	23.1 (± 2.4)	25.9 (± 2.1)	< 0.001^a^
Waist circumference – cm	87 (± 7)	100 (± 6)	< 0.001^a^
VAT – cm^2^	67 (40–93)	170 (136–235)	< 0.001^a^
Cholesterol – mg/dl	199 (± 38)	185 (± 50)	0.33
HDL – mg/dl	64 (± 21)	50 (± 14)	0.01^a^
LDL – mg/dl	117 (± 34)	109 (± 44)	0.54
Triglycerides – mg/dl	102 (± 50)	129 (± 51)	0.09
RAS inhibitors – *n* (%)	3 (18%)	20 (57%)	0.005^a^
SGLT2 inhibitors – *n* (%)	0 (0%)	1 (3%)	0.48
Lipid lowering drugs – *n*	5 (29%)	8 (23%)	0.61
Left kidney volume – cm^3^	181 (± 69)	196 (± 52)	0.51
Right kidney volume – cm^3^	151 (± 35)	181 (± 36)	0.09
Height-adjusted left kidney volume – cm^3^/m	107 (± 41)	114 (± 29)	0.63
Height-adjusted right kidney volume – cm^3^/m	93 (± 21)	105 (± 19)	0.20
eGFR at baseline – ml/min per 1.73 m^2^	74 (± 18)	78 (± 15)	0.51
eGFR at 12 months – ml/min per 1.73 m^2^	63 (± 21)	54 (± 19)	0.18
ΔeGFR – ml/min per 1.73 m^2^	12 (± 12)	24 (± 15)	0.01^a^
Relative ΔeGFR – %	16 (± 20)	31 (± 21)	0.03^a^

BMI, body mass index; BSA, body surface area; eGFR, estimated glomerular filtration rate; HDL, high density lipoprotein cholesterol; LDL, low density lipoprotein cholesterol; RAS, renin-angiotensin system; SGLT2, sodium glucose co-transporter 2; VAT, visceral adipose tissue; VO, visceral obesity. ΔeGFR, absolute change of the eGFR.

VO was defined as visceral adipose tissue area ≥ 100 cm^2^.

Gaussian-distributed parameters were displayed as means and SD, non-Gaussian distributed data as medians and interquartile ranges.

Arterial hypertension was defined as taking antihypertensive drugs.

a *P* < 0.05 in Chi-square test for categorial data, *t* test for symmetrically distributed data, and Wilcoxon rank sum test for skewed data.

### Kidney Size and Histopathological Scores

The volumetry of the nonaffected kidneys in presurgical staging CT scans showed approximately 10% higher average kidney volumes in individuals with VO than in those without VO, although this difference was not statistically significant (Table 1). Histopathological evaluation revealed mild mesangial expansion in 19% and moderate-to-severe expansion in 2% of specimens. Vascular changes such as mild and moderate-to-severe intimal thickening were present in 29% and 69% of the cohort, respectively, whereas arteriolar hyalinosis was observed in 17% (mild) and 8% (moderate-to-severe) of individuals. Interstitial fibrosis occurred in 33% (mild) and 6% (moderate-to-severe), and tubular atrophy in 29% (mild) and 6% (moderate-to-severe). These histological alterations did not differ between individuals with and without VO (Supplementary Table S2).

### The Association of VO With Morphometry

**Figure 4:**
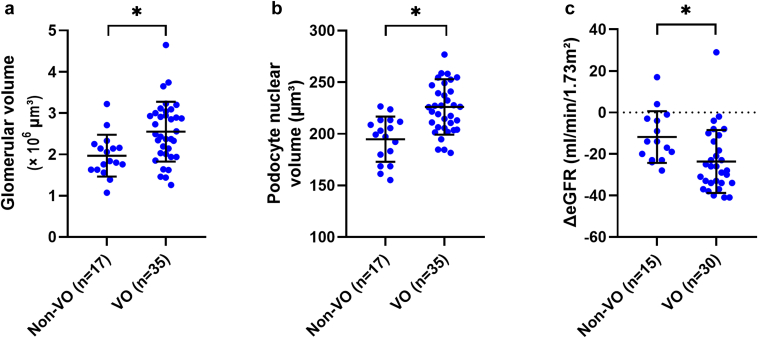
Morphometric parameters and the change in estimated glomerular filtration rate (ΔeGFR) from the prenephrectomy baseline (non–VO : 74 ± 18 ml/min per 1.73 m^2^ vs. VO : 78 ± 15 ml/min per 1.73 m^2^, *P* = 0.51) to 12-month follow-up, stratified by VO. VO was defined as visceral adipose tissue area ≥ 100 cm^2^. Panel A: glomerular volume. Panel B: podocyte nuclear volume. Panel C: ΔeGFR. Two patients died within the first 6 months after nephrectomy; 5 patients were lost to follow-up within 1 month after nephrectomy. ∗*P* < 0.05 (unpaired *t* test). VO, visceral obesity.

The specimens included a mean of 286 ± 108 nonsclerotic glomeruli per section. The data about glomerular and podocyte morphology depending on prevalent VO are displayed in Table 2. Patients with VO had larger glomerular volume than patients without VO (2.6 ± 0.7 vs. 2.0 ± 0.5 × 10^6^ μm^3^, *P* = 0.004; Figure 4). Podocyte density tended to be lower in individuals with VO than in those without VO (194 ± 50 vs. 243 ± 59 per 10^6^ μm^3^, *P* = 0.003). Larger glomerular volume was correlated with decreased podocyte density (Pearson’s r_P_: −0.41; *P* = 0.003). Subjects with VO had larger podocyte nuclei (226 ± 27 vs. 195 ± 22 μm^3^, *P* < 0.001), and a trend toward higher total podocyte nuclear volume per glomerulus (1.1 ± 0.4 vs. 0.9 ± 0.3 × 10^5^ μm^3^, *P* = 0.09) than the non-VO group. The results remained stable after adjustment for BMI and age in an analysis of covariance (Table 3) and following propensity score matching for BMI (Supplementary Table S3). Results from the nonparametric sensitivity analysis were consistent with those of the primary analysis (Supplementary Table S4).

**Table 2:** Morphometric features of patients with and without VO

Morphometric parameters	Non-VO (*n* = 17)		VO (*n* = 35)		*P*
	Mean	± SD	Mean	± SD	
Sclerotic glomeruli (%) – median (interquartile range)	0	(0–2)	1	(0–3)	0.15
Nonsclerotic glomerular count	291	(± 124)	284	(± 101)	0.81
Glomerular volume (×10^6^ μm^3^)^a^	2.0	(± 0.5)	2.6	(± 0.7)	0.004^b^
Podocyte count per glomerulus	469	(± 130)	484	(± 144)	0.73
Podocyte density (per 10^6^ μm^3^)	243	(± 59)	194	(± 50)	0.003^b^
Apparent podocyte nuclear caliper diameter, *d* (μm)	5.6	(± 0.2)	5.9	(± 0.2)	< 0.001^b^
Estimated true podocyte nuclear caliper diameter, *D* (μm)	6.7	(± 0.3)	7.0	(± 0.3)	< 0.001^b^
Correction factor for podocyte density estimation	0.411	(± 0.016)	0.396	(± 0.014)	0.001^b^
Podocyte nuclear volume (μm^3^)	195	(± 22)	226	(± 27)	< 0.001^b^
Podocyte nuclear volume per glomerulus (×10^5^ μm^3^)	0.9	(± 0.3)	1.1	(± 0.4)	0.09

VO, visceral obesity.

VO was defined as visceral adipose tissue area ≥ 100 cm^2^.

Gaussian-distributed parameters were displayed as means and SD; and non-Gaussian distributed data as medians and interquartile ranges.

a Glomerular volume was calculated with nonsclerotic glomeruli.

b *P* < 0.05 (unpaired *t* test).

**Table 3:** Sensitivity analysis: morphometric features of patients with and without VO

Morphometric parameters	Non-VO (*n* = 17)		VO (*n* = 35)		*P* for VO	*P* for BMI	*P* for age
	EMM	(95% CI)	EMM	(95% CI)			
Sclerotic glomeruli (%)	2	(0–4)	2	(1–3)	0.96	0.53	0.07
Nonsclerotic glomerular count	263	(204–322)	297	(258–336)	0.37	0.09	0.47
Glomerular volume (× 10^6^ μm^3^)^a^	1.9	(1.5–2.3)	2.6	(2.3–2.8)	0.008^b^	0.73	0.69
Podocyte count per glomerulus	467	(391–544)	485	(434–535)	0.73	0.49	0.14
Podocyte density (per 10^6^ μm^3^)	242	(213–271)	194	(175–213)	0.01^b^	0.52	0.15
Apparent podocyte nuclear caliper diameter, *d* (μm)	5.7	(5.6–5.8)	5.9	(5.8–6.0)	0.02^b^	0.15	< 0.001^b^
Estimated true podocyte nuclear caliper diameter, *D* (μm)	6.8	(6.6–6.9)	6.96	(6.87–7.04)	0.02^b^	0.10	0.002’
Correction factor for podocyte density estimation	0.406	(0.399–0.414)	0.398	(0.393–0.403)	0.11	0.08	0.12
Podocyte nuclear volume (μm^3^)	203	(192–216)	222	(214–230)	0.03^b^	0.18	0.001^b^
Podocyte nuclear volume per glomerulus (×10^5^ μm^3^)	1.0	(0.8–1.1)	1.1	(1.0–1.2)	0.33	0.32	0.73

ANCOVA, analysis of covariances; BMI, body mass index; CI, confidence interval; EMM, estimated marginal means; VO, visceral obesity.

VO was defined as visceral adipose tissue area ≥ 100 cm^2^.

Intergroup comparisons were performed using an ANCOVA, adjusted for BMI and age.

Parameters are displayed as EMM and 95% CIs.

a Glomerular volume was calculated with nonsclerotic glomeruli.

b *P* < 0.05 (ANCOVA).

Individuals with VO exhibited larger glomeruli and lower podocyte density, regardless of diabetes, hypertension, or sex, with only slightly larger podocyte nuclei in VO patients with diabetes, hypertension, or advancing age (Supplementary Tables S5–S8).

For completeness, the excluded individuals with BMI > 30 kg/m^2^ are described in Supplementary Table S9; they exhibited enlarged glomeruli, reduced podocyte density, and enlarged podocyte nuclei, concordant with the VO group. In contrast, the only difference observed between normal weight and overweight individuals (defined as BMI < 25 kg/m^2^ and BMI of 25–30 kg/m^2^) was a larger podocyte nuclear volume in overweight individuals (225 ± 26 vs. 205 ± 28 μm^3^, *P* = 0.009; Supplementary Table S10).

A subgroup of particular interest comprised individuals with normal weight and VO. Their values for glomerular volume (3.0 ± 0.7 × 10^6^ μm^3^), podocyte density (171 ± 49 per 10^6^ μm^3^), and podocyte nuclear volume (222 ± 25 μm^3^) were within similar ranges as those of the overall VO group. Because of the small sample size of normal weight individuals with VO (*n* = 11), no formal statistical analysis was performed.

### Postnephrectomy Compensation of Kidney Function

The presurgical eGFR (78 ± 15 vs. 74 ± 18 ml/min per 1.73 m^2^; *P* = 0.51) and eGFR at the 12-month follow-up showed no statistically significant differences between subjects with and without VO (54 ± 19 vs. 63 ± 21 ml/min per 1.73 m^2^; *P* = 0.18). ΔeGFR from the prenephrectomy baseline to 12 months was greater in individuals with VO (−24 ± 15 vs. −12 ± 12 ml/min per 1.73 m^2^; *P* = 0.01). Similarly, the relative ΔeGFR was more pronounced in individuals with VO (−31% ± 21% vs. −16% ± 20%; *P* = 0.03). The nonparametric sensitivity analysis showed similar results for ΔeGFR and relative ΔeGFR (*P* = 0.003 and *P* = 0.01). In an analysis of covariance, BMI and age were not associated with impaired compensation after nephrectomy (absolute and relative ΔeGFR for BMI: *P* = 0.44 and *P* = 0.68; and for age: *P* = 0.78 and *P* = 0.56); individuals with VO showed a trend toward greater ΔeGFR (*P* = 0.08), whereas the association in relative ΔeGFR was not statistically robust (*P* = 0.14).

### Structural Parameters and Renal Compensation

**Figure 5:**
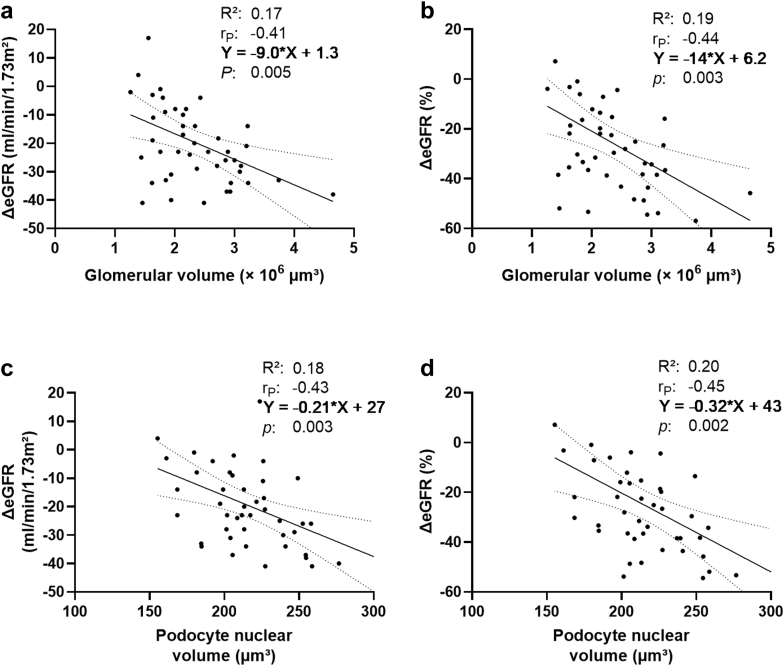
Correlation between morphometry and renal compensation. Panel A: Glomerular volume and the absolute change of the estimated glomerular filtration rate (ΔeGFR). Panel B: glomerular volume and relative ΔeGFR. Panel C: podocyte nuclear volume and ΔeGFR. Panel D: podocyte nuclear volume and relative ΔeGFR. Associations were ascertained with Pearson’s correlation coefficient (r_P_). The solid line displays the regression line; confidence bands are presented as dotted curves.

Structural parameters that were altered in VO were associated with impaired renal compensation (Table 4). Larger glomerular volume was associated with a greater decrease in ΔeGFR and relative ΔeGFR (standardized β: −0.41, *P* = 0.005 and standardized β: −0.44, *P* = 0.003; Figure 5). This corresponds to a relative ΔeGFR decline of approximately 9% (−0.44 × 22% [SD of relative ΔeGFR]) per 1 SD increase in glomerular volume (0.7 × 10^6^ μm^3^). Lower podocyte density was associated with higher ΔeGFR and relative ΔeGFR (standardized β: 0.37, *P* = 0.01 and standardized β: 0.39, *P* = 0.008; Figure 5). Larger podocyte nuclear volume was likewise associated with greater ΔeGFR and relative ΔeGFR (standardized β: −0.43, *P* = 0.003 and standardized β: −0.45, *P* = 0.002; Figure 5). The associations remained significant after adjustment for VAT, and BMI (model 1); VAT, hypertension, diabetes, age, and sex (model 2); as well as height-adjusted volume of the nonaffected kidney, and baseline eGFR (model 3); and histopathological scores (model 4, Table 4). Residual diagnostics indicated that the regression model assumptions were met. To assess the impact of potentially influential points, we repeated the regression modeling without the 1 observation with the largest residual as sensitivity analysis, which did not change the conclusions (data not shown).

**Table 4:** Multivariable linear regression analysis of structural parameters and renal compensation

Structural parameters	ΔeGFR (ml/min per 1.73 m^2^)									
	Unadjusted		Model 1		Model 2		Model 3		Model 4	
	r_P_	*P*	Standardized β	*P*	Standardized β	*P*	Standardized β	*P*	Standardized β	*P*
Glomerular volume (× 10^6^ μm^3^)	−0.41	0.005^a^	−0.38	0.009^a^	−0.43	0.007^a^	−0.47	0.002^a^	−0.43	0.008^a^
Podocytes per glomerulus	−0.09	0.57	−0.10	0.51	−0.15	0.37	−0.09	0.58	−0.09	0.58
Podocyte density (per 10^6^ μm^3^)	0.37	0.01^a^	0.31	0.04^a^	0.39	0.03^a^	0.37	0.02^a^	0.39	0.02^a^
Podocyte nuclear volume (μm^3^)	−0.43	0.003^a^	−0.37	0.03^a^	−0.56	0.005^a^	−0.48	0.001^a^	−0.45	0.005^a^
Podocyte nuclear volume per glomerulus (× 10^5^ μm^3^)	−0.25	0.10	−0.20	0.18	−0.25	0.12	−0.28	0.08	−0.25	0.12

Structural Parameters	ΔeGFR (%)									
	Unadjusted		Model 1		Model 2		Model 3		Model 4	
	r_P_	*P*	Standardized β	*P*	Standardized β	*P*	Standardized β	*P*	Standardized β	*P*
Glomerular volume (× 10^6^ μm^3^)	−0.44	0.003^a^	−0.41	0.005^a^	−0.46	0.003^a^	−0.51	0.001^a^	−0.48	0.003^a^
Podocytes per glomerulus	−0.10	0.53	−0.12	0.45	−0.18	0.28	−0.09	0.56	−0.11	0.50
Podocyte density (per 10^6^ μm^3^)	0.39	0.008^a^	0.34	0.02^a^	0.41	0.02^a^	0.41	0.009^a^	0.43	0.009^a^
Podocyte nuclear volume (μm^3^)	−0.45	0.002^a^	−0.41	0.01^a^	−0.51	0.01^a^	−0.46	0.003^a^	−0.46	0.004^a^
Podocyte nuclear volume per glomerulus (× 10^5^ μm^3^)	−0.27	0.07	−0.24	0.12	−0.28	0.08	−0.28	0.08	−0.28	0.08

eGFR, estimated glomerular filtration rate; VAT, visceral adipose tissue; ΔeGFR, absolute change of the eGFR r_P_, Pearson’s correlation coefficient.

VAT was log-transformed due to the skewed distribution.

The change in ΔeGFR was expressed as a standardized β coefficient, representing the change in SD of ΔeGFR per 1 SD of the predictor variables.

Mean and SD values of the presented parameters:

ΔeGFR: −20 ± 15 ml/min per 1.73 m^2^; relative ΔeGFR: −26% ± 22%; glomerular volume: 2.4 ± 0.7 ×10^6^ μm^3^; podocytes per glomerulus: 479 ± 139; podocyte density: 210 ± 57 per 10^6^ μm^3^; podocyte nuclear volume: 216 ± 29 μm^3^; podocyte nuclear volume per glomerulus: 1.0 ± 0.3 ×10^5^ μm^3^.

Model 1: adjusted for VAT, and body mass index.

Model 2: adjusted for VAT, hypertension, diabetes, age, and sex.

Model 3: adjusted for height-adjusted total kidney volume, and baseline eGFR.

Model 4: adjusted for histopathological scores (mesangial expansion, interstitial fibrosis, arteriolar hyalinosis, intimal thickening/fibrosis, and tubular atrophy).

a *P* < 0.05.

Absolute podocyte depletion and the total amount of podocyte nuclear volume per glomerulus (podocyte number × podocyte nuclear volume) were not associated with ΔeGFR in univariable and multivariable analysis (Table 4).

## Discussion

Our data revealed that VO in normal BMI and overweight persons is associated with glomerular and podocyte alterations. Specifically, VO was linked to enlarged glomerular volume, lower podocyte density, and podocyte nuclear hypertrophy. The association of VO with larger glomeruli is in line with previous reports, whereas podocyte alterations represent novel features in VO.^11^ We further found that VO is associated with an increased risk of impaired renal compensation at 12 months postnephrectomy. Moreover, the present report confirms the results of a previous study on renal compensation after radical nephrectomy and extends the findings to parenchymal morphology, because glomerular and podocyte features were associated with a higher ΔeGFR in our cohort.^30^

The present study specifically investigated the association of VO in normal BMI and overweight patients with glomerular morphometry and renal functional parameters, because the previous literature regarding VO had no upper BMI- threshold for inclusion.^7^^,^^11^^,^^30^ Whereas previous reports on the effects of VO on histology are scarce, an association between greater BMI and larger glomerular volume, sclerosis, and podocyte loss is well-described in the literature.^3^^,^^4^^,^^12^^,^^16^^,^^31^, ^32^, ^33^, ^34^

Notably, we did not find an association of BMI-defined overweight with glomerular and podocyte features. These findings align with a previous study by Haruhara *et al.* who divided a control group of living kidney donors by BMI status (threshold of 25 kg/m^2^) to compare glomerular and podocyte features.^4^ Consistent with our findings, the BMI-defined subgroups did not differ in glomerular volume, percentage of sclerotic glomeruli, podocyte count, and density.^4^ A screening for VO was not reported in the mentioned study.^4^

The observed glomerular enlargement in patients with VO fits with the concept of hyperfiltration.^2^^,^^35^, ^36^, ^37^, ^38^, ^39^ Glomerular hyperfiltration, characterized by an increased single nephron GFR, for example, in individuals with higher BMI, has been described in obesity-related glomerulopathy.^36^, ^37^, ^38^ The observed glomerular enlargement in subjects with VO, who showed no evidence of advanced kidney disease and thus had no clinical indication for a kidney biopsy, may represent an early stage of hyperfiltration.

In advanced stages of kidney disease, glomerular hypertrophy necessitates adaptive podocyte enlargement, which has been observed in obesity-related glomerulopathy.^2^^,^^4^^,^^35^^,^^40^ Glomerulomegaly might result in a mismatch between the glomerular tuft and total podocyte volume, leading to shear stress and podocyte detachment.^41^^,^^42^ This hypertrophic stress is reflected by increased urinary podocyte mRNA levels, even before the onset of albuminuria.^43^ Subsequent podocyte loss is a key feature of progressing glomerulosclerosis and nephron loss can be observed in individuals with manifest stages of obesity-related glomerulopathy.^2^, ^3^, ^4^^,^^35^^,^^40^ Ultimately, remnant nephrons have to compensate through further hyperfiltration.^37^

Glomerular hyperfiltration in individuals with excess body weight and VO may be related to increased tubular sodium reabsorption, reducing solute delivery to the macula densa and attenuating tubuloglomerular feedback.^2^^,^^39^^,^^44^ Additional contributors include renin-angiotensin-aldosterone system and sympathetic activation, both enhancing sodium reabsorption and systemic blood pressure.^44^^,^^45^ Hypertension is highly prevalent in subjects with obesity-related glomerulopathy and acts as an independent driver of kidney disease, promoting glomerular hypertrophy, podocyte depletion, glomerulosclerosis, and nephron loss.^3^^,^^4^^,^^16^^,^^46^^,^^47^ Moreover, podocyte stress and detachment correlate with mean arterial pressure, even within the normal range, as observed in living kidney donors without hypertension.^48^ In line with this hypothesis, the prevalence of hypertension was higher in individuals with VO. However, within the VO subgroup, neither glomerular volume nor podocyte density differed meaningfully by hypertension status, although podocyte nuclear volume was higher in individuals with hypertension. A similar pattern was observed with type 2 diabetes, a condition often linked to obesity and likewise associated with hyperfiltration, with comparable glomerular volume and podocyte density but slightly larger podocyte nuclei in affected individuals.^13^^,^^49^

The potential effect of VO on glomerular hyperfiltration raises the question of its impact on the progression of kidney disease. VO was associated with worse renal outcome in patients with CKD, with faster disease progression in autosomal dominant polycystic kidney disease and puts individuals with prediabetes at risk for nephropathy.^7^^,^^9^^,^^10^ Similar to previous studies, we observed an impaired renal compensation at the 12-month follow-up after nephrectomy in individuals with VO.^30^

Importantly, VO might affect outcomes in other populations undergoing elective nephrectomy, such as living kidney donors. Donors with VO had a lower eGFR at 12 months postnephrectomy, and VO was associated with a greater decline in ΔeGFR (whether VO persisted during follow-up remains unclear).^20^^,^^21^ This greater short-term GFR decline likely reflects an impaired ability of the remnant nephrons to increase single nephron GFR, because glomeruli that are already enlarged may have limited capacity for further adaptive hyperfiltration. Supporting this interpretation, diminished early compensatory response has been associated with lower eGFR at 5 and 10 years after unilateral nephrectomy, whether performed for donation or medical reasons.^50^^,^^51^ The long-term risk of kidney disease progression in donors with VO remains unknown, whereas obesity has been associated with long-term kidney disease progression and elevated blood pressure after donation.^33^^,^^52^^,^^53^

In our cohort, glomerulomegaly, relative podocyte loss, and podocyte nuclear volume were significantly associated with impaired renal compensation, and these associations remained robust after adjustment for clinical, radiological, and histopathological parameters. Larger glomeruli and reduced podocyte density may therefore constrain the compensatory capacity of the remnant kidney, providing a structural correlation for the observed impairment in ΔeGFR. This is particularly relevant, because lower podocyte density has been linked to progressive CKD in obesity-related glomerulopathy.^4^ Previous reports further demonstrated that enlarged glomeruli were associated with kidney disease progression,^32^^,^^33^ and lower podocyte count and density with kidney failure after nephrectomy.^17^ Taken together, VAT assessment and histological features could serve as tools to stratify the risk of kidney function decline in individuals undergoing nephrectomy.

The main limitation of our study was the relatively small sample size, which may have constrained our ability to detect small effect sizes and limited us to a descriptive assessment of additional determinants such as hypertension, diabetes, age, and sex. Nevertheless, our findings align with previous research on VO and with the pathophysiological context. Another limitation concerns the definition of VO. In the present study, VO was defined as VAT ≥ 100 cm^2^. This fixed threshold, however, does not account for differences in body height or sex, which is reflected by the fact that individuals classified with VO were more often male, whereas those without VO were more often female. Sex-specific thresholds could capture such differences; however, it remains unclear whether sex-specific cut-offs or a universal absolute threshold better reflect the biological risk of VO. Notably, men have been reported to exhibit larger glomeruli.^12^^,^^49^ This glomerular enlargement in men might reflect a higher burden of VO rather than inherent physiological sex differences. In our cohort, applying an absolute VAT threshold of 100 cm^2^ seemed to correspond to broadly comparable histological alterations in both men and women, suggesting that this cut-off may be acceptable for both sexes. Alternatively, defining VO as a proportion of the truncal cross-section may be independent of body size and sex, although this approach could be confounded by the relative contribution of other compartments such as subcutaneous adipose tissue. Nevertheless, the aim of this study was to propose a simple and clinically applicable method. VAT measured in a single cross-section has been shown to correlate with total VAT volume,^18^ and the applied threshold of 100 cm^2^ is consistent with previous studies on VO.^7^^,^^21^^,^^22^

A major strength of this study is its focus of VO in normal BMI and overweight individuals who were not selected for intrinsic kidney disease. Previous studies on VO may have been confounded by the inclusion of subjects with a BMI > 30 kg/m^2^ or by examining subjects with an established CKD who underwent indication biopsy.^7^^,^^11^^,^^30^ In contrast, we analyzed nephrectomy specimens from individuals who would not have met clinical indications for a kidney biopsy. Moreover, the use of nontumorous regions from nephrectomy specimens provided a considerable number of glomerular profiles, reducing the impact of outliers per subject compared to biopsy-based studies. Considering that previous research has primarily addressed either histological changes or renal compensation in relation to VO, an important strength of our study is the integration of both aspects.^7^^,^^11^^,^^30^^,^^45^

In conclusion, VO has significant implications on glomerular enlargement and podocyte alterations in normal BMI and overweight individuals. Excess VAT is associated with higher risk of impaired renal compensation after nephrectomy. This may be relevant for patients undergoing a planned nephrectomy, such as living kidney donors. Donor evaluations already include CT, which could be used for VAT assessments before kidney donation, particularly in individuals with a BMI < 30 kg/m^2^. Furthermore, kidney histomorphology could guide risk stratification of kidney donors.

## Disclosure

All the authors declared no competing interests.

### Funding

The study was supported by the Physician Researcher Pathway Program of the Medical University of Vienna to CP and the Vienna Science and Technology Fund (WWTF #LS20-081). The findings and conclusions in this report are those of the authors and do not necessarily represent the official position of the funding institutions.

## Supplementary Material

Supplementary File (PDF) **Figure S1.** Distribution of morphometric parameters. **Table S1.** STROBE Statement. **Table S2.** Histopathological scores of the cohort. **Table S3:** Sensitivity analysis following propensity score matching: Morphometric features of patients with and without visceral obesity (VO). **Table S4:** Nonparametric sensitivity analysis: Morphometric features of patients with and without visceral obesity (VO). **Table S5:** Morphometric features of patients with and without visceral obesity (VO), dependent on prevalent type 2 diabetes. **Table S6:** Morphometric features of patients with and without visceral obesity (VO), dependent on prevalent arterial hypertension. **Table S7:** Morphometric features of patients with and without visceral obesity (VO), dependent on patients’ sex. **Table S8:** Multivariable linear regression analysis of potential confounders and structural parameters. **Table S9:** Excluded individuals with obesity who met all inclusion criteria except the BMI requirement. **Table S10:** Morphometric features of patients with and without overweight.

## Footnotes

Supplementary File (PDF)
**Figure S1.** Distribution of morphometric parameters. **Table S1.** STROBE Statement. **Table S2.** Histopathological scores of the cohort. **Table S3:** Sensitivity analysis following propensity score matching: Morphometric features of patients with and without visceral obesity (VO). **Table S4:** Nonparametric sensitivity analysis: Morphometric features of patients with and without visceral obesity (VO). **Table S5:** Morphometric features of patients with and without visceral obesity (VO), dependent on prevalent type 2 diabetes. **Table S6:** Morphometric features of patients with and without visceral obesity (VO), dependent on prevalent arterial hypertension. **Table S7:** Morphometric features of patients with and without visceral obesity (VO), dependent on patients’ sex. **Table S8:** Multivariable linear regression analysis of potential confounders and structural parameters. **Table S9:** Excluded individuals with obesity who met all inclusion criteria except the BMI requirement. **Table S10:** Morphometric features of patients with and without overweight.
