# Supplementary material for: Visceral Adipose Tissue Alters Podometrics and Renal Compensation After Uninephrectomy
Source: Kidney Int Rep. 2025 Dec 23;11(3):103739. doi: 10.1016/j.ekir.2025.103739 (PMC12919252; doi:10.1016/j.ekir.2025.103739)

Supplementary Material

Supplementary table S1: STROBE Statement—checklist of items that should be included in reports of observational studies

	Item No.	Recommendation	Page No.
**Title and abstract**	1	(*a*) Indicate the study's design with a commonly used term in the title or the abstract	2
		(*b*) Provide in the abstract an informative and balanced summary of what was done and what was found	2
**Introduction**			
Background/rationale	2	Explain the scientific background and rationale for the investigation being reported	3
Objectives	3	State specific objectives, including any prespecified hypotheses	3
**Methods**			
Study design	4	Present key elements of study design early in the paper	4-6
Setting	5	Describe the setting, locations, and relevant dates, including periods of recruitment, exposure, follow-up, and data collection	4-5
Participants	6	(*a*) *Cohort study*—Give the eligibility criteria, and the sources and methods of selection of participants. Describe methods of follow-up<br>*Case-control study*—Give the eligibility criteria, and the sources and methods of case ascertainment and control selection. Give the rationale for the choice of cases and controls<br>*Cross-sectional study*—Give the eligibility criteria, and the sources and methods of selection of participants	4
		(*b*) *Cohort study*—For matched studies, give matching criteria and number of exposed and unexposed<br>*Case-control study*—For matched studies, give matching criteria and the number of controls per case	-
Variables	7	Clearly define all outcomes, exposures, predictors, potential confounders, and effect modifiers. Give diagnostic criteria, if applicable	4-6
Data sources/ measurement	8*	For each variable of interest, give sources of data and details of methods of assessment (measurement). Describe comparability of assessment methods if there is more than one group	4-6

Bias	9	Describe any efforts to address potential sources of bias	4-5
Study size	10	Explain how the study size was arrived at	4 + Figure 1 Panel B
Quantitative variables	11	Explain how quantitative variables were handled in the analyses. If applicable, describe which groupings were chosen and why	4 + 6
Statistical methods	12	(*a*) Describe all statistical methods, including those used to control for confounding	6
		(*b*) Describe any methods used to examine subgroups and interactions	6
		(*c*) Explain how missing data were addressed	6
		(*d*) *Cohort study*—If applicable, explain how loss to follow-up was addressed<br>*Case-control study*—If applicable, explain how matching of cases and controls was addressed<br>*Cross-sectional study*—If applicable, describe analytical methods taking account of sampling strategy	6
		(*e*) Describe any sensitivity analyses	6
**Results**			
Participants	13*	(a) Report numbers of individuals at each stage of study—eg numbers potentially eligible, examined for eligibility, confirmed eligible, included in the study, completing follow-up, and analysed	7
		(b) Give reasons for non-participation at each stage	6 + Figure 4
		(c) Consider use of a flow diagram	Figure 1 Panel B
Descriptive data	14*	(a) Give characteristics of study participants (eg demographic, clinical, social) and information on exposures and potential confounders	7
		(b) Indicate number of participants with missing data for each variable of interest	6 + Figure 4
		(c) *Cohort study*—Summarise follow-up time (eg, average and total amount)	8
Outcome data	15*	*Cohort study*—Report numbers of outcome events or summary measures over time	8
		Case-control study—Report numbers in each exposure category, or summary measures of exposure	
		Cross-sectional study—Report numbers of outcome events or summary measures	
Main results	16	(*a*) Give unadjusted estimates and, if applicable, confounder-adjusted estimates and their precision (eg, 95% confidence interval). Make clear which confounders were adjusted for	7-8

		and why they were included	
		(*b*) Report category boundaries when continuous variables were categorized	4
		(*c*) If relevant, consider translating estimates of relative risk into absolute risk for a meaningful time period	8
Other analyses	17	Report other analyses done—eg analyses of subgroups and interactions, and sensitivity analyses	7-9
**Discussion**			
Key results	18	Summarise key results with reference to study objectives	10-11
Limitations	19	Discuss limitations of the study, taking into account sources of potential bias or imprecision. Discuss both direction and magnitude of any potential bias	11-12
Interpretation	20	Give a cautious overall interpretation of results considering objectives, limitations, multiplicity of analyses, results from similar studies, and other relevant evidence	11-12
Generalisability	21	Discuss the generalisability (external validity) of the study results	12
**Other information**			
Funding	22	Give the source of funding and the role of the funders for the present study and, if applicable, for the original study on which the present article is based	13

Supplementary table S2: Histopathological scores of the cohort.

	Non-VO (n=17)	VO (n=35)	
**Histopathological scores**	**No. (%)**	**No. (%)**	***P***
Mesangial expansion			0.10
- none	15 (88%)	26 (74%)	
- mild	1 (6%)	9 (26%)	
- intermediate/severe	1 (6%)	0 (-)	
Intimal thickening/fibrosis			0.11
- none	0 (-)	1 (3%)	
- mild	8 (47%)	7 (20%)	
- intermediate/severe	9 (53%)	27 (77%)	
Interstitial fibrosis			0.94
- none	11 (65%)	21 (60%)	
- mild	5 (29%)	12 (34%)	
- intermediate/severe	1 (6%)	2 (6%)	
Tubular atrophy			0.84
- none	12 (71%)	22 (63%)	
- mild	4 (23%)	11 (31%)	
- intermediate/severe	1 (6%)	2 (6%)	
Arteriolar hyalinosis			0.61
- none	13 (76%)	26 (74%)	
- mild	2 (12%)	7 (20%)	
- intermediate/severe	2 (12%)	2 (6%)	

Legend: Data were presented as counts and percentages. Percentages were rounded to the next full number. Visceral obesity (VO) was defined as visceral adipose tissue area ≥100 cm².
Histopathological alterations were scored semi-quantitatively from between none, mild (°I), intermediate (°II), and severe (°III) lesions. Wilcoxon rank sum test was performed for statistical analysis.

Supplementary table S3: Sensitivity analysis following propensity score matching: Morphometric features of patients with and without visceral obesity (VO).

	Non-VO (n=12)		VO (n=12)		
**Morphometric Parameter**	**Mean**	**±SD**	**Mean**	**±SD**	***P***
Sclerotic glomeruli (%) – median (interquartile range)	1	(0-2)	1	(0-2)	0.95
Non-sclerotic glomerular count	314	(±98)	255	(±68)	0.10
**Glomerular volume ($\times10^6$ µm³)#**	**2.0**	**(±0.5)**	**2.9**	**(±0.7)**	**0.002***
Podocyte count per glomerulus	464	(±152)	514	(±152)	0.43
**Podocyte density (per 10^6 µm³)**	**237**	**(±50)**	**183**	**(±47)**	**0.01***
Apparent podocyte nuclear caliper diameter *d* (µm)	5.7	(±0.2)	5.9	(±0.2)	0.03*
Estimated true podocyte nuclear caliper diameter *D* (µm)	6.7	(±0.2)	6.9	(±0.2)	0.01*
Correction factor for podocyte density estimation	0.410	(±0.011)	0.398	(±0.014)	0.045*
**Podocyte nuclear volume (µm³)**	**197**	**(±21)**	**218**	**(±22)**	**0.03***
Podocyte nuclear volume per glomerulus ($\times10^5$ µm³)	0.9	(±0.3)	1.1	(±0.4)	0.17

Legend: Visceral obesity (VO) was defined as visceral adipose tissue area ≥100 cm².
Propensity score matching based on BMI was used to generate comparable groups of individuals with VO and without VO.
Gaussian-distributed parameters were displayed as means and standard deviations (SD), non-Gaussian distributed data as medians and interquartile ranges.
#Glomerular volume was calculated with non-sclerotic glomeruli.
*P<0.05 (unpaired t-Test).

Supplementary table S4: Non-parametric sensitivity analysis: Morphometric features of patients with and without visceral obesity (VO).

	**Non-VO (n=17)**		**VO (n=35)**		
**Morphometric Parameter**	**Mean**	**±SD**	**Mean**	**±SD**	***P***
Sclerotic glomeruli (%) – median (interquartile range)	0	(0-2)	1	(0-3)	0.15
Non-sclerotic glomerular count	291	(±124)	284	(±101)	0.99
**Glomerular volume ($\times 10^6$ µm³)#**	**2.0**	**(±0.5)**	**2.6**	**(±0.7)**	**0.004***
Podocyte count per glomerulus	469	(±130)	484	(±144)	0.54
**Podocyte density (per 10^6 µm³)**	**243**	**(±59)**	**194**	**(±50)**	**0.006***
Apparent podocyte nuclear caliper diameter *d* (µm)	5.6	(±0.2)	5.9	(±0.2)	<0.001*
Estimated true podocyte nuclear caliper diameter *D* (µm)	6.7	(±0.3)	7.0	(±0.3)	<0.001*
Correction factor for podocyte density estimation	0.411	(±0.016)	0.396	(±0.014)	0.002*
**Podocyte nuclear volume (µm³)**	**195**	**(±22)**	**226**	**(±27)**	**<0.001***
Podocyte nuclear volume per glomerulus ($\times 10^5$ µm³)	0.9	(±0.3)	1.1	(±0.4)	0.09

Legend: Visceral obesity (VO) was defined as visceral adipose tissue area ≥100 cm².
Gaussian-distributed parameters were displayed as means and standard deviations (SD), non-Gaussian distributed data as medians and interquartile ranges.
#Glomerular volume was calculated with non-sclerotic glomeruli.
**P*<0.05 (Mann Whitney U test).

Supplementary table S5: Morphometric features of patients with and without visceral obesity (VO), dependent on prevalent type 2 diabetes.

	**Non-VO (n=17)**		**VO without T2D (n=25)**		**VO with T2D (n=10)**	
**Morphometric Parameter**	**Mean**	**±SD**	**Mean**	**±SD**	**Mean**	**±SD**
Sclerotic glomeruli (%) – median (interquartile range)	0	(0-2)	1	(0-3)	2	(0-4)
Non-sclerotic glomerular count	291	(±124)	262	(±97)	337	(±96)
**Glomerular volume (×10^6 µm³)#**	**2.0**	**(±0.5)**	**2.5**	**(±0.8)**	**2.6**	**(±0.6)**
Podocyte count per glomerulus	469	(±130)	479	(±141)	496	(±160)
**Podocyte density (per 10^6 µm³)**	**243**	**(±59)**	**197**	**(±55)**	**188**	**(±35)**
Apparent podocyte nuclear caliper diameter *d* (µm)	5.6	(±0.2)	5.9	(±0.2)	6.0	(±0.3)
Estimated true podocyte nuclear caliper diameter *D* (µm)	6.7	(±0.3)	7.0	(±0.2)	7.1	(±0.3)
Correction factor for podocyte density estimation	0.411	(±0.016)	0.396	(±0.014)	0.397	(±0.015)
**Podocyte nuclear volume (µm³)**	**195**	**(±22)**	**221**	**(±23)**	**239**	**(±33)**
Podocyte nuclear volume per glomerulus (×10^5 µm³)	0.9	(±0.3)	1.1	(±0.3)	1.2	(±0.4)

Legend: Visceral obesity (VO) was defined as visceral adipose tissue area ≥100 cm².
Gaussian-distributed parameters were displayed as means and standard deviations (SD), non-Gaussian distributed data as medians and interquartile ranges.
#Glomerular volume was calculated with non-sclerotic glomeruli.

Supplementary table S6: Morphometric features of patients with and without visceral obesity (VO), dependent on prevalent arterial hypertension.

	Non-VO without HTN (n=9)		Non-VO with HTN (n=8)		VO without HTN (n=9)		VO with HTN (n=26)	
**Morphometric Parameter**	**Mean**	**±SD**	**Mean**	**±SD**	**Mean**	**±SD**	**Mean**	**±SD**
Sclerotic glomeruli (%) – median (interquartile range)	0	(0-2)	1	(0-3)	0	(0-3)	2	(0-3)
Non-sclerotic glomerular count	332	(±150)	245	(±70)	314	(±84)	273	(±106)
**Glomerular volume ($\times 10^6$ µm³)#**	**1.8**	**(±0.6)**	**2.1**	**(±0.4)**	**2.6**	**(±0.9)**	**2.5**	**(±0.7)**
Podocyte count per glomerulus	453	(±153)	487	(±105)	495	(±134)	478	(±150)
**Podocyte density (per 10^6 µm³)**	**248**	**(±58)**	**238**	**(±63)**	**202**	**(±70)**	**191**	**(±42)**
Apparent podocyte nuclear caliper diameter *d* (µm)	5.5	(±0.2)	5.8	(±0.1)	5.8	(±0.3)	6.0	(±0.2)
Estimated true podocyte nuclear caliper diameter *D* (µm)	6.5	(±0.3)	6.8	(±0.1)	6.8	(±0.3)	7.1	(±0.2)
Correction factor for podocyte density estimation	0.416	(±0.018)	0.405	(±0.010)	0.402	(±0.012)	0.394	(±0.014)
**Podocyte nuclear volume (µm³)**	**180**	**(±19)**	**211**	**(±11)**	**211**	**(±29)**	**231**	**(±24)**
Podocyte nuclear volume per glomerulus ($\times 10^5$ µm³)	0.8	(±0.3)	1.0	(±0.2)	1.0	(±0.4)	1.1	(±0.4)

Legend: Visceral obesity (VO) was defined as visceral adipose tissue area ≥100 cm².
Gaussian-distributed parameters were displayed as means and standard deviations (SD), non-Gaussian distributed data as medians and interquartile ranges.
#Glomerular volume was calculated with non-sclerotic glomeruli.

Supplementary table S7: Morphometric features of patients with and without visceral obesity (VO), dependent on patients' sex.

	Non-VO (Male) (n=2)		Non-VO (Female) (n=15)		VO (Male) (n=27)		VO (Female) (n=8)	
**Morphometric Parameter**	**Mean**	**±SD**	**Mean**	**±SD**	**Mean**	**±SD**	**Mean**	**±SD**
Sclerotic glomeruli (%) – median (interquartile range)	1	(0-1)	0	(0-2)	2	(0-3)	1	(0-4)
Non-sclerotic glomerular count	380	(±1)	279	(±128)	305	(±98)	212	(±81)
**Glomerular volume (×10^6 µm³)#**	**1.9**	**(±0.4)**	**2.0**	**(±0.5)**	**2.6**	**(±0.7)**	**2.5**	**(±0.8)**
Podocyte count per glomerulus	391	(±25)	480	(±135)	477	(±148)	506	(±138)
**Podocyte density (per 10^6 µm³)**	**210**	**(±27)**	**248**	**(±61)**	**190**	**(±50)**	**207**	**(±48)**
Apparent podocyte nuclear caliper diameter *d* (µm)	5.3	(±0.1)	5.7	(±0.2)	5.9	(±0.2)	5.9	(±0.2)
Estimated true podocyte nuclear caliper diameter *D* (µm)	6.3	(±0.0)	6.7	(±0.2)	7.0	(±0.3)	7.0	(±0.2)
Correction factor for podocyte density estimation	0.417	(±0.002)	0.410	(±0.016)	0.396	(±0.014)	0.396	(±0.014)
**Podocyte nuclear volume (µm³)**	**165**	**(±5)**	**199**	**(±20)**	**226**	**(±28)**	**226**	**(±25)**
Podocyte nuclear volume per glomerulus (×10^5 µm³)	0.6	(±0.1)	1.0	(±0.3)	1.1	(±0.4)	1.1	(±0.3)

Legend: Visceral obesity (VO) was defined as visceral adipose tissue area ≥100 cm².
Gaussian-distributed parameters were displayed as means and standard deviations (SD), non-Gaussian distributed data as medians and interquartile ranges.
#Glomerular volume was calculated with non-sclerotic glomeruli.

Supplementary table S8: Multivariable linear regression analysis of potential confounders and structural parameters.

**Morphometric Parameter**	**Standardized β**	***P***
Glomerular volume (×10^6 µm³)#		
- Age	-0.07	0.64
- Diabetes	0.04	0.77
- Hypertension	0.03	0.84
- Sex	0.004	0.98
Podocyte density (per 10^6 µm³)		
- Age	-0.19	0.17
- Diabetes	-0.06	0.66
- Hypertension	-0.09	0.52
- Sex	0.19	0.25
Podocyte nuclear volume (µm³)		
- Age	**0.45**	**<0.001**
- Diabetes	0.25	0.052
- Hypertension	**0.40**	**0.001**
- Sex	0.13	0.43

Legend: For glomerular volume, podocyte density, and podocyte nuclear volume, four separate multivariable models were run, each adjusted for visceral obesity (defined as visceral adipose tissue area ≥100 cm²) and one additional parameter: age, prevalent type 2 diabetes, hypertension, or sex.

Supplementary table S9: Excluded individuals with obesity who met all inclusion criteria except the BMI requirement.

	**Patients with obesity (n=14)**	
<u>**Morphometric Parameter**</u>	**Mean**	**±SD**
Sclerotic glomeruli (%) – median (interquartile range)	1	(0-2)
Non-sclerotic glomerular count	280	(±156)
Glomerular volume ($\times 10^6$ µm³)#	2.7	(±0.7)
Podocyte count per glomerulus	539	(±227)
Podocyte density (per 10^6 µm³)	200	(±69)
Apparent podocyte nuclear caliper diameter *d* (µm)	5.8	(±0.3)
Estimated true podocyte nuclear caliper diameter *D* (µm)	6.9	(±0.3)
Correction factor for podocyte density estimation	0.407	(±0.010)
**Podocyte nuclear volume (µm³)**	**217**	**(±37)**
Podocyte nuclear volume per glomerulus ($\times 10^5$ µm³)	1.1	(±0.4)

Legend: Obesity was defined as body mass index >30 kg/m².
Visceral obesity (defined as visceral adipose tissue area ≥100 cm²) was present in 13 individuals. Gaussian-distributed parameters were displayed as means and standard deviations (SD),
non-Gaussian distributed data as medians and interquartile ranges.
#Glomerular volume was calculated with non-sclerotic glomeruli.

Supplementary table S10: Morphometric features of patients with and without overweight.

	**Normal weight (n=24)**		**Overweight (n=28)**		
**Morphometric Parameter**	**Mean**	**±SD**	**Mean**	**±SD**	***P***
Sclerotic glomeruli (%) – median (interquartile range)	1	(0-2)	2	(0-3)	0.15
Non-sclerotic glomerular count	295	(±121)	279	(±97)	0.61
Glomerular volume ($\times 10^6$ µm³)[#]	2.4	(±0.8)	2.3	(±0.6)	0.72
Podocyte count per glomerulus	475	(±143)	483	(±137)	0.85
Podocyte density (per 10^6 µm³)	209	(±63)	211	(±53)	0.92
Apparent podocyte nuclear caliper diameter *d* (µm)	5.7	(±0.3)	5.9	(±0.2)	0.007*
Estimated true podocyte nuclear caliper diameter *D* (µm)	6.8	(±0.3)	7.0	(±0.3)	0.005*
Correction factor for podocyte density estimation	0.407	(±0.015)	0.396	(±0.015)	0.02*
**Podocyte nuclear volume (µm³)**	**205**	**(±28)**	**225**	**(±26)**	**0.009***
Podocyte nuclear volume per glomerulus ($\times 10^5$ µm³)	1.0	(±0.4)	1.1	(±0.3)	0.28

Legend: Overweight was defined as body mass index 25-30 kg/m².
Gaussian-distributed parameters were displayed as means and standard deviations (SD), non-Gaussian distributed data as medians and interquartile ranges.
[#]Glomerular volume was calculated with non-sclerotic glomeruli.
**P*<0.05 (unpaired t-Test).

Supplementary figure S1: Distribution of morphometric parameters. Panel A: Glomerular volume. Panel B: Podocyte count. Panel C: Podocyte density. Panel D: Podocyte nuclear volume. Panel E: Total podocyte nuclear volume per glomerulus.

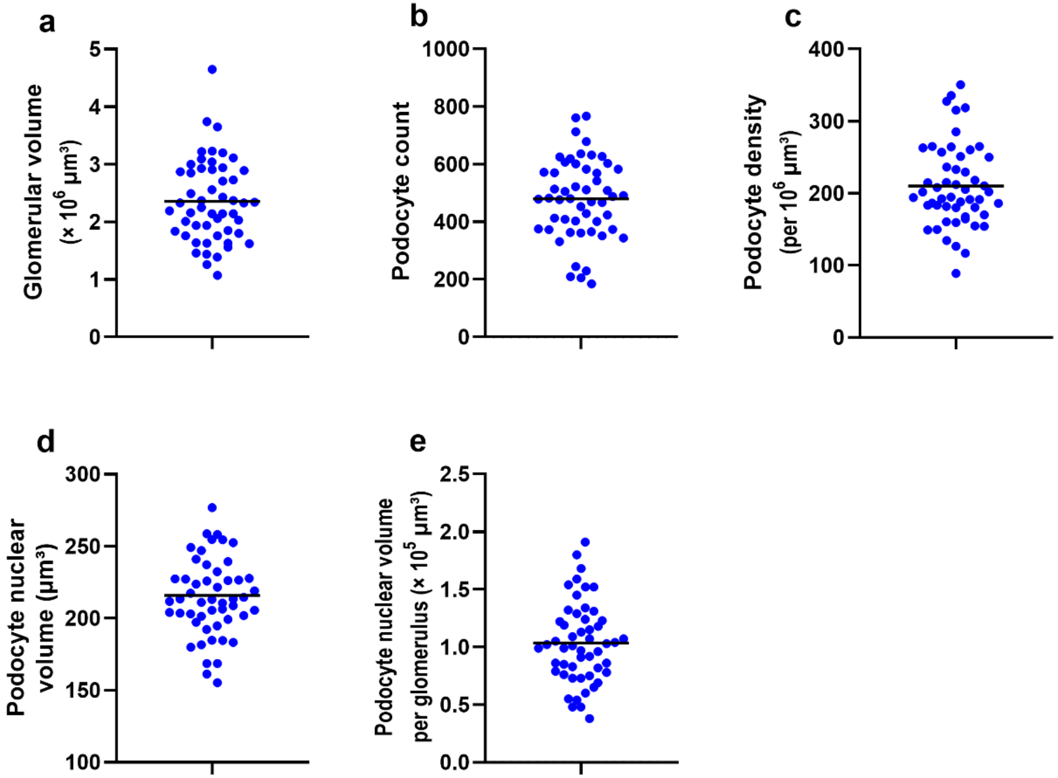

Supplement: Supplementary File (PDF) — Figure S1. Distribution of morphometric parameters. Table S1. STROBE Statement. Table S2. Histopathological scores of the cohort. Table S3: Sensitivity analysis following propensity score matching: Morphometric features of patients with and without visceral obesity (VO). Table S4: Nonparametric sensitivity analysis: Morphometric features of patients with and without visceral obesity (VO). Table S5: Morphometric features of patients with and without visceral obesity (VO), dependent on prevalent type 2 diabetes. Table S6: Morphometric features of patients with and without visceral obesity (VO), dependent on prevalent arterial hypertension. Table S7: Morphometric features of patients with and without visceral obesity (VO), dependent on patients’ sex. Table S8: Multivariable linear regression analysis of potential confounders and structural parameters. Table S9: Excluded individuals with obesity who met all inclusion criteria except the BMI requirement. Table S10: Morphometric features of patients with and without overweight. [file mmc1.pdf]
